# Case report: Sepsis secondary to infected protracted parotid sialocele after maxillofacial oncologic surgery in a dog

**DOI:** 10.3389/fvets.2024.1382546

**Published:** 2024-05-01

**Authors:** Stephanie Goldschmidt, Jamie Anderson, Janny Evenhuis, Eric Stoopler, Thomas P. Sollecito

**Affiliations:** ^1^Department of Surgical and Radiologic Sciences, School of Veterinary Medicine, University of California, Davis, Davis, CA, United States; ^2^Department of Oral Medicine, School of Dental Medicine, University of Pennsylvania, Philadelphia, PA, United States

**Keywords:** sialocele, sepsis, oncologic surgery, oral surgery, parotid gland, parotid duct, Stensen’s duct

## Abstract

An 8-year-old male intact mixed breed dog was treated for a 3.7×3×3.6 cm grade 1 multilobular osteochondrosarcoma (MLO) arising from the dorsal aspect of the right coronoid process with a coronoidectomy, a zygomectomy, and a caudal maxillectomy. Ten months later, the dog presented for a swelling near the right angular process, which was presumed to be a locoregional recurrence. Blood work and initial staging tests (abdominal ultrasound) had mild abnormalities of no clinical concern/significance. The dog was hospitalized with a plan for computed tomographic (CT) scan of skull and chest the following day. Overnight, the swelling rapidly increased, and the dog became laterally recumbent, febrile, and hypotensive. Laboratory evaluation revealed hypoglycemia, elevated lactate, and elevated band neutrophils with moderate toxicity, most consistent with sepsis. The dog was stabilized with fluid resuscitation, intravenous (IV) antibiotics, IV dextrose, and pressor support. Once stabilized, a contrast CT scan was performed, which revealed evidence of an infected parotid gland sialocele. To our knowledge, this is the first veterinary case that describes sepsis secondary to an infected protracted parotid sialocele.

## Introduction

Major maxillofacial oncologic surgery incurs multiple perioperative risks, including hemorrhage, infection, dehiscence, and functional complications. However, most of the complications are reported in the immediate perioperative period (0–3 months), with <2% being reported as short-term (3–6 months), mid-term (6–12 months), or long-term (>12 months) sequela (1). Here, we report an iatrogenic parotid sialocele developing 10 months following caudal oncologic surgery in a dog. Parotid duct stenosis and secondary dilation have been previously reported as a delayed complication in the veterinary literature occurring 2–3 years following caudal maxillectomy (2, 3). In both previous cases, there were no secondary complications related to the salivary gland disease. In the reported case, secondary infection of a sialocele resulted in sepsis, a ramification that has never been documented to occur with a chronic untreated sialocele.

Sepsis is broadly defined as a systemic inflammatory response to infection (4–7). This clinically manifests as two or more of the following: (1) change in temperature (hyper or hypothermia), (2) tachycardia, (3) tachypnea, and/or (4) an abnormal leukogram (leukocytosis, leukopenia, or high band neutrophil count) (4). When cardiovascular instability and profound metabolic/cellular abnormalities are superimposed on sepsis, it is termed septic shock, which carries a higher mortality risk than sepsis alone. In companion animals, infection from the gastrointestinal tract is the most common source of sepsis and septic shock (4–6).

Conversely, sepsis related to oral infection is exceedingly rare. In humans, sepsis has been described as secondary to odontogenic infection with a reported incidence of 3.3% (16/483 patients with odontogenic abscess over a 5-year period) (8). All septic dogs included in this cohort also had pre-existing immunosuppression or risk factors for infection, such as endocrine disease or nicotine abuse. There is only one report in companion animals that describes sepsis occurring from oral infection in a cat (9). The reported cat developed diabetic ketoacidosis as part of the disease progression, suggesting diabetes mellitus acted as a risk factor for septic progression. Although there is no consensus in the literature for humans or companion animals, it appears that either local or systemic immune suppression may be a prerequisite for septic progression from an oral source. This is the first report in the literature of septic progression from a sialocele in any species.

## Case description

**Figure 1:**
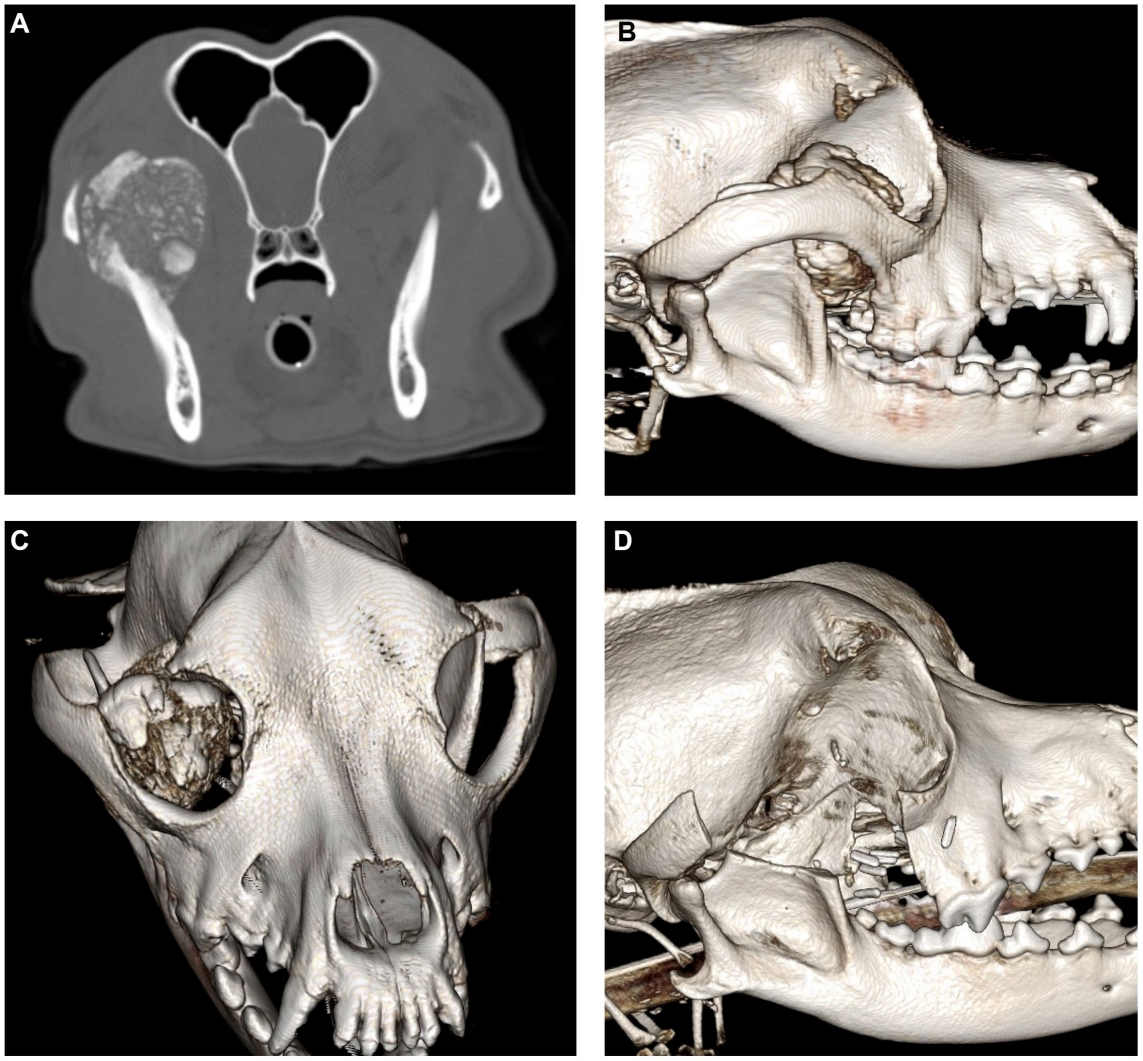
Contrast-enhanced CT images of the initial MLO prior to surgical removal viewed in the axial multiplanar reconstruction **(A)** and three-dimensional (3D) reconstructions **(B,C)**. Post-operative cone beam CT images **(D)**.

An 8-year-old male intact mixed breed dog was presented to the Dentistry and Oral Surgery Service (DOSS) at the University of California, Davis, for presumptive recurrent multilobular osteochondrosarcoma (MLO). The dog had been treated 10 months prior for a 3.7×3×3.6 cm Grade 1 MLO arising from the dorsal aspect of the right coronoid process (Figure 1) with a coronoidectomy, a zygomectomy, and a caudal maxillectomy. The parotid duct/gland was not directly visualized during the surgical procedure. However, no special efforts were made to ensure the gland or duct was easily visualized intraoperatively, such as cannulating the duct and instilling with methylene blue (10). Post-surgical margins were incomplete, and radiation therapy was recommended but declined by the owner.

On presentation, there was an approximately 6-cm soft, non-painful, fluctuant swelling on the right lateral portion of the face near the angular process of the mandible. The remainder of the physical examination was normal. The pulse and respiratory rate were within normal limits. Rectal temperature was not obtained due to the hyperactivity and objection of the dog. Other than the previously diagnosed MLO, there were no comorbidities, and the patient was not on any medications. A complete blood count (CBC) revealed mild leukopenia at 5730/μL (6,000-13,000) with no other specific cell lines below or above the reference range. The chemistry panel was within normal limits. Initial staging with abdominal ultrasound showed prostatic changes most consistent with benign prostatic hyperplasia, chronic renal changes, and mild cholecystic debris.

The dog was admitted to the hospital 4 days later with a plan to complete locoregional staging with anesthetized head, neck, and thoracic computed tomography (CT) the following day. Overnight, the facial swelling rapidly increased, and the dog became febrile (104.2°), laterally recumbent, and hypotensive (pulse quality too poor for accurate Doppler reading). A point-of-care blood gas panel (ABU 800 Flex blood gas analyzer, Brea, CA) was performed revealing hypoglycemia at 56 mg/dL (reference: 64–123) and elevated lactate at 3.4 mmol/L (reference: <2.5) with no other overt electrolyte abnormalities. CBC/chemistry was then performed (5 days post initial clinical pathology examination). Chemistry revealed moderate hyponatremia at 136 mmol/L (reference: 143–151), mild hypochloremia at 102 mmol/L (reference: 108–116), low bicarbonate at 13 mmol/L (reference: 20–29), hypocalcemia at 9.2 mg/dL (reference: 9.6–11.2, likely secondary to low protein), hypoalbuminemia at 2.7 g/dL (reference: 3.4–4.3), mild hyperglobulinemia at 3.2 g/dL (reference: 1.7–3.1), and mild elevation of the AST at 65 μL (reference: 20–49), CK at 903 IU/L (reference: 55–257), and ALKP at 146 IU/L (reference: 14–91). CBC revealed the presence of toxic band neutrophils at 848/μL (reference: 0–0), though the neutrophil count was within the normal range at 5,623/μL (3,000-10,500). Based on the presence of pyrexia, severe hypotension, hypoglycemia, elevated lactate, and the presence of toxic band neutrophils, the dog was diagnosed with presumptive sepsis (Table 1; Supplementary Table S1).

**Table 1:** Temporal changes in CBC or chemistry from initial presentation to development of presumed sepsis.

	8/10/23	8/15/23
Chemistry		
Anion Gap (mmol/L)	21 (H)	25 (H)
Sodium (mmol/l)	147	136 (L)
Potassium (mmol/l)	4.6	3.8
Chloride (mmol/L)	111	102 (L)
Bicarbonate (mmol/L)	20	13 (L)
Phosphorus (mg/dL)	3.6	4.6
Calcium (mg/dL)	10.2	9.2 (L)
bun (mg/dL)	14	18
creatinine (mg/dL)	0.9	1.1
Glucose (mg/dL)	108	121 (H *supplemented)
Total protein (g/dL)	6.9	5.9
albumin (g/dL)	3.6	2.7 (L)
globulin (g/dL)	3.3 (H)	3.2 (H)
ALT (IU/L)	34	23
AST (IU/L)	28	65 (H)
Creatine kinase (IU/L)	177	903 (H)
Alkaline phosphatase (IU/L)	44	146 (H)
GGT (IU/L)	<3	<3
Cholesterol (mg/dL)	352	313
Bilirubin total (mg/dL)	<0.2	0.2
Magnesium (mg/dL)	1.7 (L)	1.5 (L)
Hematology		
RBC (m/μL)	6.01	6.02
Hb (gm/dL)	14.5	14.6
Hematocrit (%)	42.6	41.7
MCV (fl)	70.8	69.2
MCH (pg)	24.2	24.2
MCHC (gm/dL)	34.2	34.9
RDW (%)	13.0	12.8
Nucleated RBCs (/100 WBC)		6
Anisocytosis	Slight	Slight
Polychromasia	Rare	Rare
Schistocytes		Rare
Acanthocytes		Rare
Echinocytes		Moderate
Blister cells		Rare
WBC (/μL)	5,730 (L)	9,460
WBC (Corrected, /μL)		8,925
BANDS* (/μL)		848 (H)
*Comment		Moderate toxicity, rare marked toxicity
Neutrophils* (/μL)	3,870	5,623
*Comment		Moderate toxicity, rare marked toxicity
Lymphocytes (/μL)	1,360	1785
Monocytes (/μL)	370	446
Eosinophils (/μL)	110	179
Basophils (/μL)	30	45
Platelets* (/μL)	367,000	218,000
*Comment	Clumps seen	Clumps seen, rare macroplatelets
MPV (fl)	11.0	12.6
Plasma protein (gm/dL)	8.0	7.1

Abnormal values are shaded and marked as high (H) or low (L).

The patient was stabilized by the Critical Care service with resuscitative IV isotonic crystalloid boluses, 5% dextrose supplementation, and IV antibiotics (ampicillin sulbactam at 30 mg/kg Q8h and enrofloxacin at 10 mg/kg Q24h). Treatment with pressor agents was not required following initial fluid resuscitation. After 8 h of resuscitative treatment, the dog was deemed hemodynamically and metabolically stable enough for general anesthesia to investigate and treat the suspected source of infection. This decision was based on the sustained mean arterial blood pressure > 60 mm Hg, normal EKG, normal glucose, improved mentation, and decreasing lactate (Table 1). The dog’s right-sided facial swelling actively progressed during the hours of resuscitative treatment, advancing rostrally to the muzzle and also crossing midline to the ventral cervical region.

**Figure 2:**
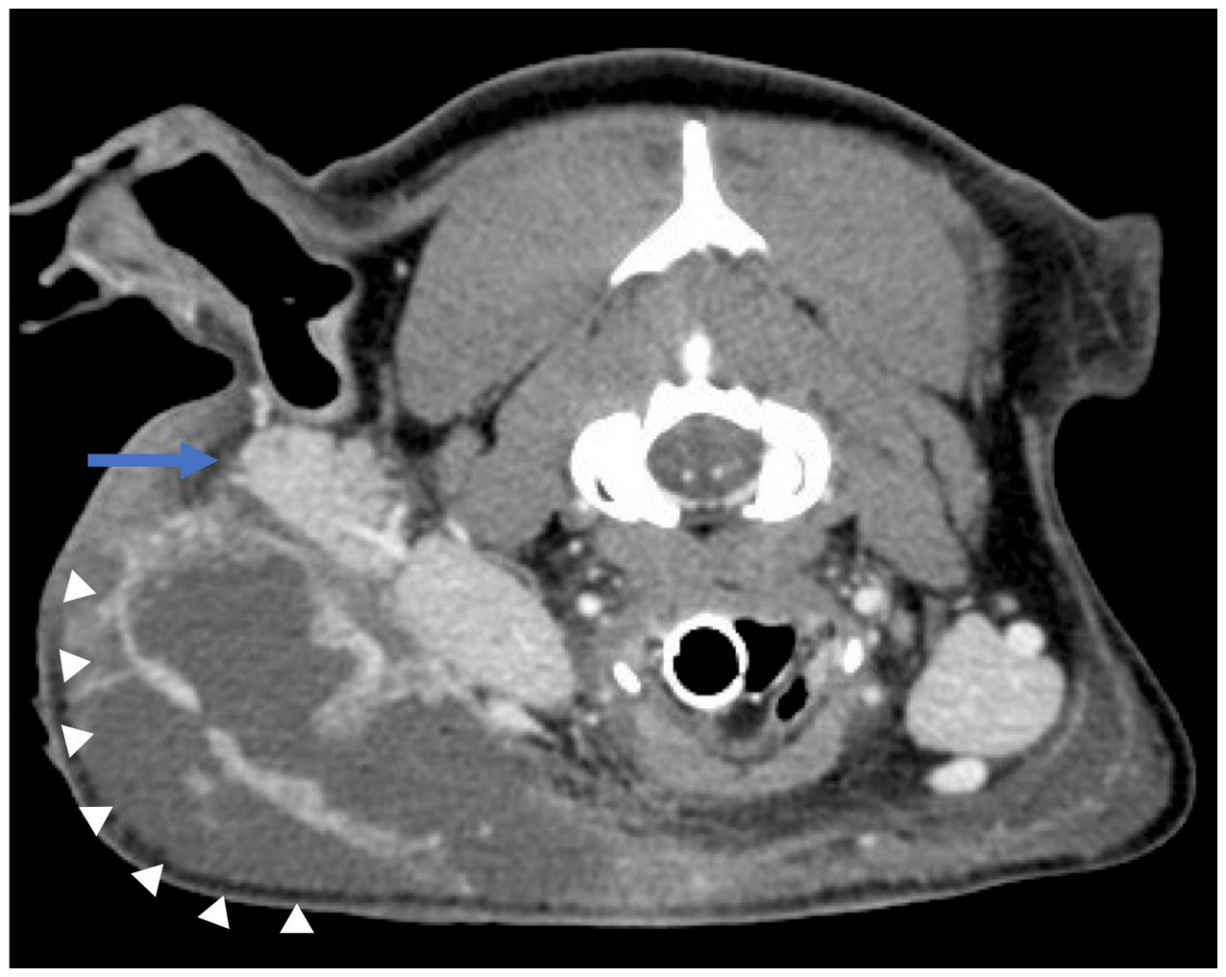
Contrast-enhanced CT images of the head viewed in the axial plane showing a multiloculated region of fluid accumulation with peripheral contrast enhancement (white arrowheads) tracking to the parotid salivary gland (blue arrow) on the side of the previous surgery.

Once anesthetized, nor-epinephrine was required to maintain a mean arterial blood pressure > 60 mm Hg. CT scan of the head revealed a multiloculated region of fluid accumulation with peripheral contrast enhancement tracking to the parotid salivary gland on the side of the previous surgery. The fluid tracked to a defect in the overlying skin. Additionally, the region of fluid accumulation resulted in secondary compression of the right maxillary, linguofacial, and external jugular veins. There was also evidence of recurrence of the previously resected MLO at the ostectomy site of the right coronoid process. Differential diagnosis included local abscessation and cellulitis, but the final assessment was most consistent with an infected sialocele originating from the parotid salivary duct (Figure 2).

Right parotid gland sialoadenectomy with debridement of infected/necrotic tissue and resection of ulcerated and necrotic skin was performed to remove the nidus of infection. The region of MLO recurrence of the right coronoid process was also resected. The salivary gland, samples of necrotic tissue, and right coronoid process fragment were submitted in buffered formalin for histopathologic analysis. A closed-suction Jackson–Pratt drain was placed. Culture and sensitivity of purulent material grew small numbers of *Streptococcus canis* as well as abundant *Bacteroides* and *Prevotella* species. The patient was hospitalized for continued supportive care post-surgery, which included pain control (fentanyl constant rate infusion 3-5mcg/kg/h, carprofen 2.2 mg/kg IV), IV crystalloid therapy, and continuation of IV antibiotics. A repeat blood gas analysis revealed decreasing lactate, normal glucose, and no new biochemical abnormalities (Supplementary Table S1). No repeat CBC or chemistry panel was performed. While in the hospital, the patient maintained bilateral palpebral reflex and facial tone. The Jackson–Pratt drain was removed, and the patient was discharged 48 h postoperatively. Antibiotic therapy with amoxicillin with clavulanic acid at 13 mg/kg PO Q12h and enrofloxacin at 10 mg/kg PO Q12h were appropriate based on culture and sensitivity and were continued for a total of 4 weeks postoperatively without any secondary complications.

There was minimal (approximately 2 cm) dehiscence at the center of the skin surgical site noted at the 2-week recheck examination, which was most likely secondary to tension on closure after extensive necrotic skin resection; otherwise, the patient healed with no major complications and did not require revision surgery. The biopsy results revealed severe inflammation and thrombosis of the connective tissue surrounding the salivary gland with relatively unremarkable salivary tissue. The resected coronoid process confirmed the presence of MLO recurrence with complete resection. Further rechecks 1 month and 3 months later revealed no recurrence of the swelling or septic progression after discontinuing antibiotic therapy. However, there is continued self-trauma to the site presumed to be secondary to neuralgia or pruritus from fibrous tissue remodeling. The dog is currently being treated with gabapentin (10 mg/kg TID) and amantadine (3 mg/kg SID), which effectively controls the clinical signs of self-trauma under direct supervision. The Elizabethan collar is still worn when the dog is not directly monitored by the owners.

## Discussion

Salivary gland disease is very rare in dogs, with a reported incidence of 0.3% (11). Sialocele, defined as an accumulation of saliva in the fascial planes secondary to leakage from the salivary gland or duct, is the most common salivary disease in dogs (11–13). The parotid salivary glands are less commonly affected than the mandibular and sublingual salivary glands (10–13). Furthermore, direct trauma to the parotid gland/duct appears to play a more central role in the development of the parotid salivary gland sialocele compared to the mandibular salivary gland (14–16). In an experimental dog model, direct trauma and/or ligation of the mandibular gland or duct did not result in sialocele development (17). Conversely, most cases of parotid sialocele in dogs reference trauma, either iatrogenic or otherwise, as the source (2, 3, 14–16).

Iatrogenic trauma from caudal oncologic surgery resulting in parotid sialocele is extremely rare. In fact, there is nearly a 10-year gap since this was previously reported in the literature. It is unclear whether this complication is underreported or whether the incidence is truly this low. Comparatively, in humans, iatrogenic parotid sialocele is reported at an approximately 10% post-surgical complication rate when direct surgery of the parotid gland is performed (18, 19). Sialocele formation secondary to damage of the parotid duct during other maxillofacial surgeries is rare with no epidemiologic data available (20–22). Iatrogenic sialocele formation is always reported in the immediate perioperative period. Sialocele development secondary to traumatic injury also primarily presents within weeks of trauma (23) with isolated reports of protracted sialocele development (24).

**Figure 3:**
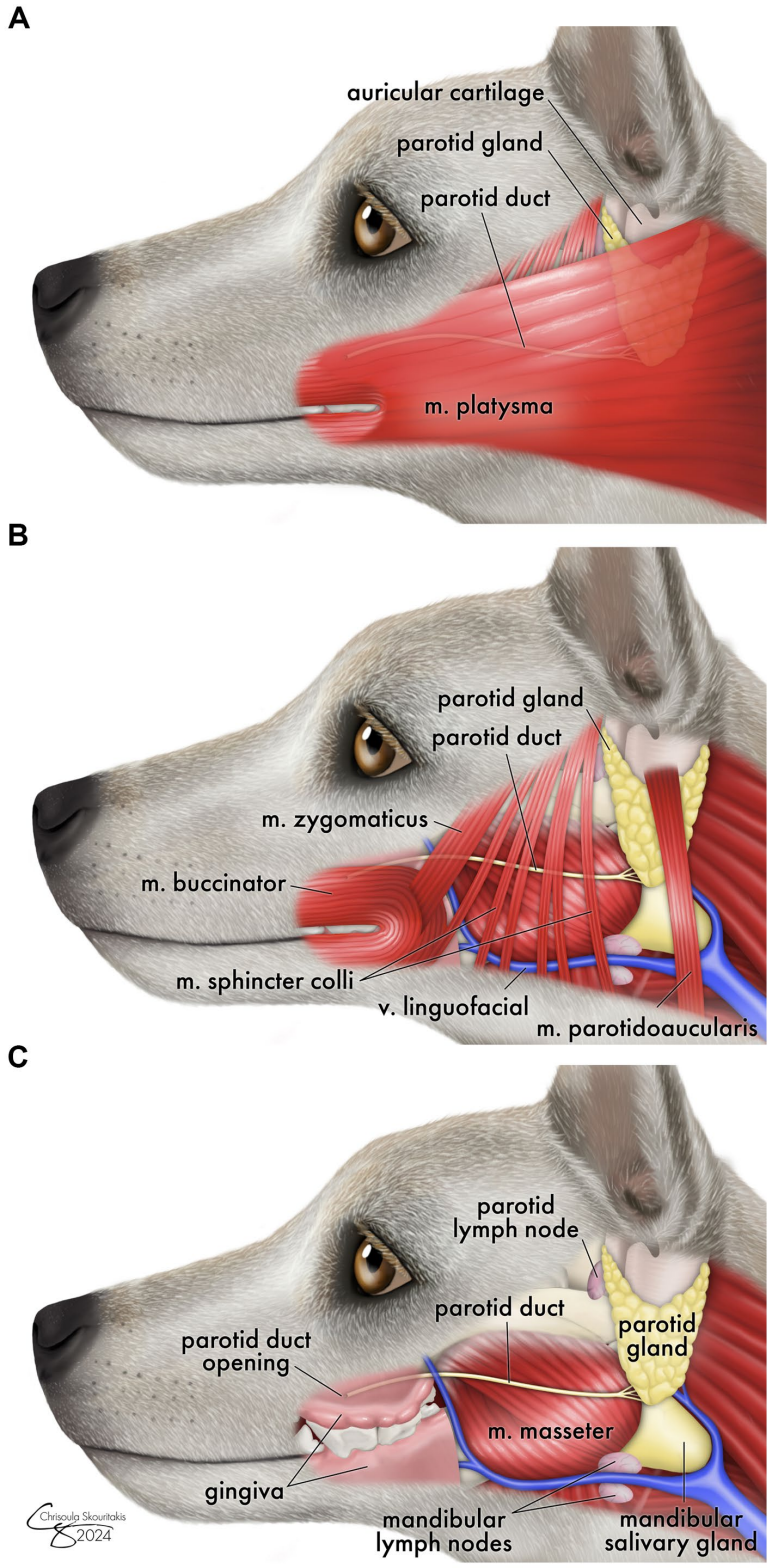
Schematic drawing showing the anatomic location of the parotid salivary duct as it runs from the parotid gland over the surface of the masseter muscle to the parotid papilla. Surrounding musculature (m.) is shown moving from most superficial **(A)** - deep **(C)**. Relevant major blood vessels and lymph nodes are shown for anatomic reference.

The mechanism for protracted sialocele development in dogs and why this differs from the traditional timeline of iatrogenic sialocele in humans is unclear. Presumably, during caudal surgery in the three reported canine cases, there was direct damage to the parotid duct, which travels from the parotid gland across the masseter to release at the parotid papilla (Figure 3). Surgery of the dorsocaudal maxilla, masseter muscles, or coronoid process has a risk of iatrogenic trauma to the duct.

Theoretically, direct damage could result in a slow leakage of saliva from the duct, which may take months to years to present as a clinical sialocele. However, immediate salivary leakage would have likely interfered with surgical healing due to the robust inflammatory response that occurs secondary to saliva extravasation (11, 12). Furthermore, although there are no published data on the rate of saliva production in dogs, or the expected expansion rate of a sialocele, assuming dogs produce saliva at a rate similar to humans (0.3–0.4 mL/min) (25), it is unlikely that it would take years for a clinical sialocele to develop. Accordingly, most recurrent sialoceles, which are presumed to be due to remaining glandular tissue and direct saliva extravasation, recur within 3–6 months of initial surgery (26, 27). Conversely, a recent article in dogs reported recurrent sialoceles developed at a median of 3 years following sialoadenectomy (28).

Alternatively, protracted iatrogenic sialoceles may occur secondary to post-surgical fibrosis and remodeling causing parotid duct stenosis, duct dilation, and eventual rupture. It is unclear why stenosis would not result in complete or partial parotid gland atrophy, which is reported in cats, rabbits, and rats with experimental parotid duct ligation (29–31). Potentially, in most dogs, atrophy is the clinical course, explaining the rarity of iatrogenic sialocele development post caudal maxillofacial surgery. Ideally, in both the current and historical cases, a sialogram would have been performed to elucidate the status of the duct and better explain the clinical course of the disease. Mechanistic information could help better prevent this complication during surgery.

Furthermore, although it is documented that a chronic sialocele can theoretically become secondarily infected (13), subjectively infection is clinically rare and to the authors’knowledge has never been reported in the literature. The dearth of infected sialocele reports may be due to the antimicrobial and immune properties of saliva (32–34). Salivary glands have resident B cells, T cells, macrophages, and dendritic cells, enabling them to be an integral part of both the innate and adaptive immune response (32, 33) in the oral cavity. Furthermore, salivary peroxidases, most notably lactoperoxidase, can generate hypothiocyanous acid, which has direct oxidative antimicrobial effects (34).

This is the first report of any species of secondary sepsis from an infected sialocele. At the authors’ institutions, these are not treated as urgent cases, unless they present in a pharyngeal location. However, this report suggests that a sialocele should potentially be triaged as a higher priority, due to the potential for sepsis if secondary infection occurs. This dog was already admitted to the hospital when sepsis occurred allowing for rapid intervention and positive outcome. It has been shown that administration of broad-spectrum antibiotic therapy as soon as possible (ideally within 1 h), appropriate fluid resuscitation, and source control of infection are associated with decreased risk of mortality (6, 7). In hindsight, further investigation of the initial leukopenia is warranted to elucidate why there may have been a low white cell count, as this likely represented the consumption of cells secondary to infection. At the time, workup for the low cell count was not prioritized based on the remainder of the physical examination and laboratory evaluation being normal.

This case highlights the risk of parotid sialocele formation as a rare long-term complication with caudal maxillofacial surgery. Further care should be taken to visualize the parotid duct and gland during caudal maxillary surgery to prevent iatrogenic damage to this structure. Aids to visualization of the parotid duct could be in the form of a preoperative sialogram at the planning CT scan or, additionally, the use of cannulation with methylene blue intraoperatively (10). Additionally, this case highlights the potential for severe systemic complications, such as sepsis, to occur with salivary gland disease. Early intervention for sialoceles is recommended to prevent severe complications.

## Data availability statement

The raw data supporting the conclusions of this article will be made available by the authors, without undue reservation.

## Ethics statement

Written informed consent was obtained from the owners for the participation of their animals in this study.

## Author contributions

SG: Writing – original draft, Writing – review & editing, Conceptualization, Data curation. JA: Writing – review & editing, Conceptualization. JE: Data curation, Writing – review & editing. ES: Conceptualization, Writing – review & editing. TS: Conceptualization, Writing – review & editing.
