# Supplementary material for: Case report: Sepsis secondary to infected protracted parotid sialocele after maxillofacial oncologic surgery in a dog
Source: Front Vet Sci. 2024 May 1;11:1382546. doi: 10.3389/fvets.2024.1382546 (PMC11094748; doi:10.3389/fvets.2024.1382546)
Supplement: Supplementary file 1 [file Table_1.DOCX]

**Table S1:** Temporal metabolic and chemical changes based on serial blood gas panels (ABU 800 Flex blood gas analyzer, Brea CA) while the patient was hospitalized. The earliest time point is on the left of the table. Repeat panels were taken approximately every 2 hours.

|  | 8/15 | 8/15 | 8/15 | 8/15 | 8/15 | 8/15 | 8/15 | 8/15 | 8/15 | 8/16 | 8/16 | 8/16 |
| --- | --- | --- | --- | --- | --- | --- | --- | --- | --- | --- | --- | --- |
|  |  |  |  |  |  |  |  |  |  |  |  |  |
| Sample Type | VEN | ART | ART | ART | ART | ART | ART | ART | ART | ART | ART | VEN |
| BODY TEMP (°F) | 98.6 | 103.0 | 98.6 | 98.6 | 96.7 | 98.6 | 98.5 | 99.9 | 99.9 | 98.6 | 98.6 | 98.6 |
| Fraction of Inspired Oxygen (%) | 21.0 | 21.0 | 21.0 | 100.0 | 100.0 | 21.0 | 100.0 | 100.0 | 21.0 | 21.0 | 21.0 | 21.0 |
| pH (PH U) | 7.353 | 7.359 | 7.412 | 7.328 | 7.300 | 7.184 | 7.185 | 7.254 | 7.328 | 7.353 | 7.400 | 7.404 |
| pCO2 (mmHG) | 27.9 | 27.4 | 24.2 | 28.6 | 33.8 | 48.8 | 46.7 | 40.1 | 34.2 | 36.5 | 32.8 |  |
| pO2 (mmHG) | 29.7 | 92.8 | 104 | 477 | 435 | 438 | 412 | 466 | 102 | 96.9 | 98.7 | 123 |
| HCO3- (MM/L) | 15.1 | 15.0 | 15.1 | 14.6 | 16.1 | 17.7 | 17.0 | 17.1 | 17.4 | 19.8 | 19.9 |  |
| CO2 TOTAL (MM/L) | 16.0 | 15.9 | 15.8 | 15.5 | 17.2 | 19.1 | 18.4 | 18.4 | 18.5 | 20.9 | 20.9 |  |
| BASE DEFICIT/BASE EXCESS (MM/L) | -9.3 | -9.4 | -8.7 | -10.2 | -9.0 | -9.2 | -9.8 | -8.7 | -7.4 | -4.8 | -4.1 |  |
| SODIUM (MEQ/L) | 142 | 139 | 140 | 139 | 139 | 139 | 139 | 138 | 139 | 141 | 143 | 145 |
| POTASSIUM (MEQ/L) | 3.1 | 3.3 | 3.4 | 2.8 | 3.1 | 3.2 | 3.4 | 3.8 | 4.1 | 3.8 | 3.8 | 4.3 |
| IONIZED  CALCIUM (MM/L) | 1.11 | 1.35 | 1.22 | 1.22 | 1.29 | 1.36 | 1.28 | 1.32 | 1.40 | 1.41 | 1.29 | 1.34 |
| CHLORIDE (MEQ/L) | 114 | 112 | 114 | 114 | 113 | 112 | 115 | 114 | 112 | 115 | 117 | 117 |
| GLUCOSE (MG/DL) | 56 | 85 | 88 | 118 | 142 | 165 | 163 | 162 | 123 | 124 | 119 | 154 |
| LACTATE (mmol/L) | 3.4 | 2.4 | 2.0 | 2.7 | 2.4 | 2.1 | 1.5 | 1.7 | 2.1 | 0.9 | 0.6 | 0.7 |
| CREATININE (mg/dL) | 1.03 | 0.98 | 0.91 | 0.97 | 0.89 | 0.94 | 0.82 | 0.87 | 1.02 | 0.73 | 0.68 | 0.74 |
| Anion Gap (mmol/L) | 15.1 | 16.2 | 14.5 | 13.4 | 13.6 | 12.4 | 11.0 | 11.0 | 13.5 | 10.3 | 9.3 |  |
